# Clinical outcomes of Najuta thoracic stent graft system for arch aneurysms

**DOI:** 10.3389/fsurg.2023.1167714

**Published:** 2023-05-16

**Authors:** Yasunori Iida, Takashi Hachiya, Hidetoshi Oka, Yu Inaba, Takahisa Miki, Hideyuki Shimizu

**Affiliations:** ^1^ Department of Cardiovascular Surgery, Saiseikai Yokohamashi Tobu Hospital, Yokohama, Japan; ^2^ Department of Cardiovascular Surgery, Keio University, Tokyo, Japan

**Keywords:** Najuta thoracic stent graft system, thoracic endovascular aortic repair, fenestrated stent graft, bird-beak configuration, semicustom-made device

## Abstract

**Objectives:**

We aimed to elucidate the perioperative and short-term clinical outcomes of the Najuta thoracic stent graft system with fenestrations for supra-aortic vessels.

**Methods:**

We retrospectively investigated the perioperative and short-term clinical outcomes of 20 patients treated for arch or distal arch aneurysms using the Najuta thoracic stent graft system during the period from May 2019 to February 2023.

**Results:**

The technical success rate of the Najuta thoracic stent graft system was 95%. Of the 20 patients, 17 patients (85.0%) underwent concomitant extra-anatomical supra-aortic bypass. Postoperative CT revealed type Ia (*n* = 2) and type II (*n* = 3) endoleaks which disappeared on follow-up. The postoperative complications were stroke (*n* = 2, 10.0%), paraplegia (*n* = 1, 5.0%), and paraparesis (*n* = 1, 5.0%). In a very old patient, a blood transfusion was performed from the common iliac artery using the retroperitoneal approach. There were no aorta-related complications such as retrograde type A dissection or distal stent graft–induced new entry.

**Conclusions:**

We treated arch or distal arch thoracic aneurysms by inserting a tube-type stent graft as a scaffold on the peripheral site and placing the Najuta thoracic stent graft on the proximal site. By extending the landing zone to Zone 0 and using a low radial force, which is a feature of the Najuta thoracic stent graft system, postoperative bird-beak and aorta-related complications were avoided. The treatment of arch and distal arch aortic aneurysms using the Najuta thoracic stent graft system showed acceptable perioperative and short-term clinical outcomes. Thoracic endovascular aortic repair using the Najuta thoracic stent graft system may be a potential treatment option for arch and distal arch aortic aneurysms, warranting further studies.

## Introduction

Thoracic endovascular aortic repair (TEVAR) has been the treatment of choice for aortic arch pathology because of the short landing zone and aortic arch curvature. However, TEVAR occasionally produces a “bird-beak” configuration and has a poor outcome (1, 2).

Various stent graft systems have been approved for use with TEVAR. In Japan, the Najuta thoracic stent graft system (SB-Kawasumi Laboratories, Inc., Kanagawa, Japan) was approved for clinical use in January 2013 (3, 4). This stent is a semicustom-made device composed of a self-expandable stainless steel Z-stent and a thin expanded polytetrafluoroethylene graft. Since its approval, clinical experiences of an endovascular arch and distal arch aneurysm repair using the Najuta thoracic stent graft system have been reported (5–8). In fact, the Najuta thoracic stent graft system was given the Conformité Européenne (CE) mark in Europe in 2017. An instructor qualification system for using the Najuta thoracic stent graft system is currently in place in Japan.

Our experience with the Najuta thoracic stent graft system for the treatment of arch aneurysms yielded acceptable clinical outcomes. We were also able to develop a surgical method that compensates for the limitations of the Najuta thoracic stent graft system and takes advantage of its strengths for the treatment of a saccular arch aneurysm protruding toward the arch’s lesser curvature.

In this study, we aimed to elucidate the perioperative and short-term clinical outcomes of the Najuta thoracic stent graft system with fenestrations for supra-aortic vessels.

## Patients and methods

### Ethical statement

The study protocol was approved by the Institutional Review Board of Saiseikai Yokohamashi Tobu Hospital on January 6, 2022 (approval number: 20210175). Written informed consent was obtained from each patient.

### Patient characteristics and study design

We investigated 20 patients in this study consisting of 15 men and 5 women. The mean age of the patients was 79.5 ± 6.1 years (range, 71–94 years). The characteristics of the patients are shown in Table 1. These patients were treated for arch or distal arch aneurysms using the Najuta thoracic stent graft system. We used a retrospective study design wherein we retrospectively reviewed and evaluated the clinical outcomes of these patients.

**Table 1:** Characteristics of patients (*n* = 20).

Variable	Number of patients (%)
Age (years)	79.5 ± 6.1
Men	15 (75.0)
Hypertension	20 (100.0)
COPD	6 (30.0)
Diabetes	4 (20.0)
Dyslipidemia	14 (70.0)
CKD	5 (25.0)
Ischemic heart disease	5 (25.0)
Cerebral vascular disease	6 (30.0)
Hyperuricemia	3 (15.0)
Aneurysm shape	
Saccular	13 (65.0)
Saccular with CBAD	1 (5.0)
Fusiform	4 (20.0)
PAU	2 (10.0)
Shaggy aorta	2 (10.0)
Aneurysm location	
Anterior wall	13 (65.0)
Lesser curvature	5 (25.0)
Greater curvature	2 (10.0)
Previous open-heart surgery	1 (5.0)
Previous EVAR	1 (5.0)
Previous open repair for AAA	1 (5.0)
Diameter (mm)	52.7 ± 8.0

Data are expressed as mean ± SD or *n* (%).

COPD, chronic obstructive pulmonary disease; CKD, chronic kidney disease; CBAD, chronic type B aortic dissection; PAU, penetrating atherosclerotic ulcer; EVAR, endovascular aortic repair; AAA, abdominal aortic aneurysm.

### Preparation of Najuta thoracic stent graft system and patient inclusion criteria

The Najuta thoracic stent graft system is a semicustom-made fenestrated graft. The position of its fenestration can be matched to preserve the arch vessels according to the aortic arch shape for each case. The pre-shape of the stent graft can be optimized for each case by changing the connection position of each stent to approximate the three-dimensional (3D) flexion of the aortic arch.

The preparation of the Najuta stent graft system involves three procedures. *First*, thin-slice computed tomography (CT) of 2 mm or less is performed, and then the *Digital Imaging and Communications in Medicine* (DICOM) data are sent to SB-Kawasumi Laboratories, Inc. (Tokyo, Japan). *Second*, the Najuta thoracic stent graft is prepared, and the recommended placement report is provided to the surgeon. *Third*, a “deployment simulation report” is created from the CT data using a 3D printer assuming that the stent graft is deployed in the aorta.

The inclusion criteria were as follows: patients with proximal and distal neck lengths ≥20 mm and patients with proximal and distal aortic diameters ≥20 and <38 mm. Patients with an arch aneurysm involving the arch vessels, an isolated distal arch aneurysm, and acute type B aortic dissection were excluded from this study.

### Procedure

All TEVAR patients were administered general anesthesia at our institution to ensure sufficient respiratory arrest for the precise deployment of the fenestrated stent graft. We exposed the unilateral femoral artery as the access site of the stent graft.

For the “pull-through technique,” we inserted a 0.032 in × 400 cm guidewire (Radifocus, TERUMO, Tokyo, Japan) from the right brachial artery (BA). We caught the guidewire in the ascending aorta with a 27–45 mm snare catheter (Atrieve, Argon Medical Devices, Frisco, TX, United States) that was inserted from the femoral artery.

We performed digital subtraction angiography to ensure alignment of the peripheral fenestration marker made in the Najuta thoracic stent graft with the peripheral marker made in the left common carotid artery (LCA) or left subclavian artery (LSA). We recommend the deployment of the Najuta thoracic stent graft under self-heartbeat. For deployment, we held the Najuta thoracic stent graft rod firmly with the right hand and pulled down the outer sheath with the left hand. When the proximal two or three stents were released, the blood pressure pushed the Najuta thoracic stent graft to the periphery. We pushed back the Najuta thoracic stent graft system with the right hand, and the endoskeleton of the Najuta thoracic stent graft was fully expanded and deployed by the blood pressure. After removing the fixation thread at the tip of the Najuta thoracic stent graft, we slowly pulled out the inner sheath. The most important point when collecting the proximal tip of the Najuta thoracic stent graft system is to be careful not to let the proximal tip get into the endoskeleton of the Najuta thoracic stent graft and interfere with the fins for migrating the Najuta thoracic stent graft to the peripheral side.

For aneurysms that cannot be treated with one piece of the Najuta thoracic stent graft, we usually place a second Najuta stent graft on the peripheral side. When another stent graft that is manufactured by another company is placed before the Najuta thoracic stent graft, we additionally place a fenestrated Najuta thoracic stent graft to extend the proximal neck after obtaining a certain degree of proximal neck shield with the distal stent graft. In this way, a more therapeutic effect is expected. We provided a representative procedural Supplementary Video.

### Follow-up, end points, and definitions

We followed up the patients in our outpatient clinic at 1 week, 3 months, 6 months, and 12 months after the operation and annually thereafter. The follow-up protocols consisted of physical findings, laboratory examinations, and contrast-enhanced CT.

The end points included technical success, postoperative complications, aneurysm shrinkage rate, overall survival rate, freedom from reintervention rate, and freedom from aorta-related event rate.

The aortic aneurysm shrinkage rate was obtained by subtracting the aneurysm diameter on a recent CT from the preoperative diameter and dividing the outcome by the preoperative aneurysm diameter. The definitions of clinical terms related to TEVAR procedures are in accordance with the reporting standards suggested by the Society for Vascular Surgery (9).

### Statistical analysis

Values were expressed as mean ± SD for continuous variables and frequencies or proportions for categorical variables. The overall survival rate and freedom from reintervention rate were obtained by the Kaplan–Meier method using SPSS Statistics v.24 (IBM Corp. released 2016; IBM SPSS Statistics for Windows v.24, Armonk, NY, United States).

## Results

A total of 20 patients were treated by TEVAR using the Najuta thoracic stent graft system. The mean postoperative follow-up period was 22.3 ± 11.7 (4–44) months. The procedural data and postoperative data of the patients are shown in Tables 2 and 3, respectively.

**Table 2:** Procedural data and complications.

Variable	Number of patients (%)
Technical success	19 (95.0)
Proximal landing zone	
Zone 0	20 (100.0)
Concomitant procedure	
RSA-LSA bypass	10 (50.0)
RSA-LCA-LSA bypass	5 (25.0)
LSA embolization	10 (50.0)
Operation time (min)	168.2 ± 43.1
Blood transfusion	1 (5.0)
Complications	
Stroke	2 (10.0)
Paraplegia	1 (5.0)
Paraparesis	1 (5.0)
BA pseudoaneurysm	1 (5.0)
AKI	0 (0)
In-hospital mortality	0 (0)

Data are expressed as mean ± SD or *n* (%).

RSA, right subclavian artery; LCA, left common carotid artery; LSA, left subclavian artery; BA, brachial artery; AKI, acute kidney injury.

**Table 3:** Postoperative data.

Variable	Number of patients (%)
Endoleak	5 (25.0)
Type Ia	2 (10.0)
Type II	3 (15.0)
Aneurysm shrinkage rate (%)	11.9
Additional procedures	3 (15.0)
LSA embolization for type II endoleak	2 (10.0)
Repair of the BA pseudoaneurysm	1 (5.0)
Distal end of the stent graft (Th level)	7.6 ± 1.3 (6–12)
Hospital stay (days)	12.7 ± 8.8 (6–37)
Follow-up duration (months)	19.9 ± 11.4 (2–41)

Data are expressed as mean ± SD or *n* (%).

LSA, left subclavian artery; BA, brachial artery.

Technical success, which was defined as successful deployment in the intended position of the aortic arch, was achieved in 19 patients (95.0%). In one patient, the second stent graft caught the “fin” of the first stent graft. Thus, we discontinued the deployment of the second stent graft. The proximal landing zone was Zone 0 in all 20 patients.

**Figure 1:**
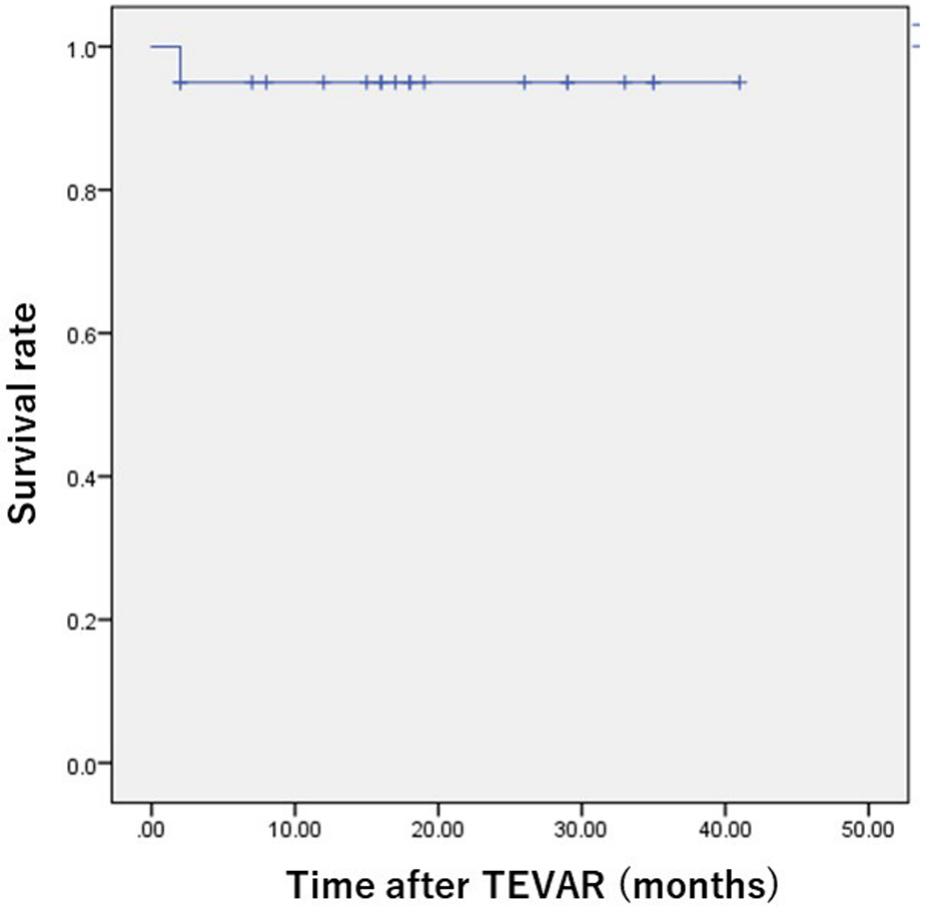
Kaplan–Meier overall survival curve in patients who underwent TEVAR using the Najuta thoracic stent graft system. The survival rate was 95.0% 24 months postoperatively.

Regarding the concomitant procedures, 17 of the 20 patients (85.0%) underwent debranching bypass. LSA embolization was performed in 11 patients (55.0%), with additional LSA embolization in two patients because of type II endoleak 8 and 47 days after the first procedure. In this series, two patients (10.0%) with a shaggy aorta in the aortic arch suffered from stroke: one patient (5.0%) suffered from paraplegia, and the other patient (5.0%) suffered from paraparesis. One patient suffered from a pseudoaneurysm of the BA and underwent surgical repair on the puncture site. The other patients were discharged without major postoperative complications. The overall survival rate was 95.0% at 1, 12, and 24 months (Figure 1).

**Figure 2:**
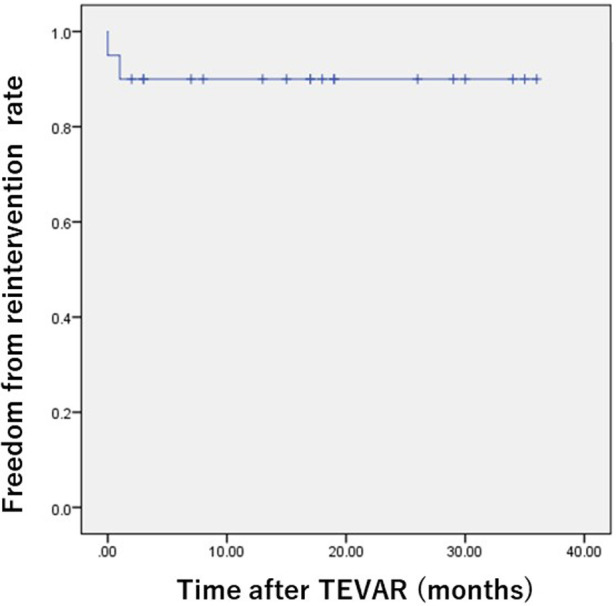
Kaplan–Meier freedom from reoperation rate after TEVAR using the Najuta thoracic stent graft system. The freedom from reoperation rate was 90.0% 24 months postoperatively.

Type 1a endoleak occurred in two patients (10.0%), and type II endoleak occurred in three patients (15.0%). In the patients with type Ia endoleak, meticulous follow-up was conducted, and strict blood pressure control reduced the degree of leakage. In the patients with type II endoleak from the LSA, two patients underwent LSA embolization using an Amplatzer Vascular Plug (St. Jude Medical Inc., St. Paul, MN, United States), but the other patient did not undergo additional treatment as the endoleak was not observed during the follow-up. The freedom from reintervention rates were 95.0%, 90.0%, and 90.0% at 1, 12, and 24 months, respectively (Figure 2).

The positions of the distal end of the stent graft were at T6 (two patients: 10.0%), T7 (10 patients; 50.0%), T8 (six patients; 30.0%), T9 (one patient: 5.0%), and T12 (one patient; 5.0%), with an average of T7.6 ± 1.3. Of the 20 patients, 11 patients (55.0%) were treated with two stent grafts, and two patients (10.0%) were treated with three stent grafts. Of these 13 patients, distal stent grafts made by other companies were used.

Aortic events such as enlargement, retrograde aortic dissection (RTAD), and distal stent graft–induced new entry were not observed during the follow-up period.

## Discussion

In this study, we set out to elucidate the perioperative and short-term clinical outcomes of the Najuta thoracic stent graft system with fenestrations for supra-aortic vessels. The use of this system with TEVAR showed no postoperative bird-beak and aorta-related complications, indicating its potential as a treatment option for arch and distal arch aortic aneurysms.

Since the approval of the Najuta thoracic stent graft system for clinical use in Japan in January 2013, treatments of arch and distal arch aneurysms by TEVAR have expanded in terms of anatomical indications. For the treatment of arch aneurysms, total arch replacement is the recommended gold standard surgical procedure because of its stable long-term results. However, since the introduction of TEVAR for the treatment of thoracic aortic aneurysms or dissections, TEVAR has reduced the number of serious complications such as respiratory failure and renal dysfunction compared with the gold standard total arch replacement, as well as markedly improved postoperative quality of life and shortened hospital stay (10). Thus, the number of thoracic aortic pathology cases treated by TEVAR has seen an increase in recent years (11).

Nevertheless, TEVAR has also some limitations for treating aortic arch aneurysms. These include the inability to secure a sufficient proximal neck length because of the positional relationship with the supra-aortic branches and the inability to obtain a good attachment between the lesser curvature and the stent graft (i.e., bird-beak configuration) which hinders proper sealing. To extend the proximal neck, the debranching and chimney procedures have been developed. However, supra-aortic debranching TEVAR reportedly caused a stroke in 7% of patients (0%–25%) as the most common perioperative complication (12). Concerns about graft-related complications at a later period also leave questions about supra-aortic branch bypass for younger patients.

In the present patient series, postoperative stroke was recognized in 2 of 17 patients (11.8%) with the supra-aortic branch bypass. However, the degree and symptoms of the postoperative stroke were not permanent, and improvement was achieved by postoperative rehabilitation. We were able to extend the proximal neck length with the supra-aortic branch bypass and avoid the bird-beak phenomenon in Zone 2. This allowed the Najuta thoracic stent graft to be inserted into Zone 0. As a procedure to avoid the supra-aortic branch bypass, the chimney technique is used for reconstructing the arch vessels without debranching. However, the chimney technique presents concerns about the gutter leak and RTAD that occur from the gap between the stent grafts in Zone 0, as well as the risk of developing a stroke. Moreover, the chimney technique has the disadvantage of being a complicated procedure compared with debranching.

**Figure 3:**
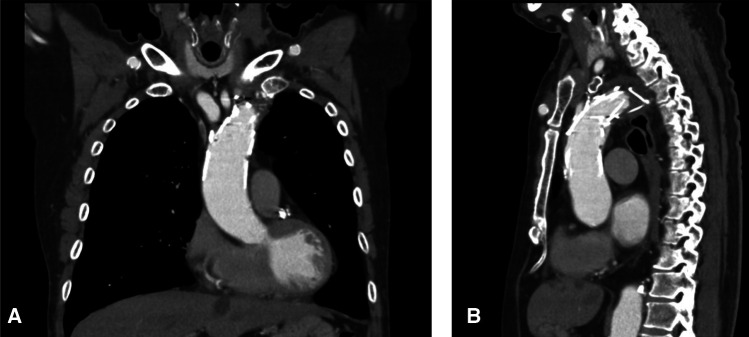
Postoperative CT showed no bird-beak phenomenon both in the coronal (**A**) and sagittal (**B**) sections.

The Najuta thoracic stent graft system is the first commercial fenestrated thoracic stent graft system developed in Japan. The procedure for using the system involves the selection of the metal skeleton that most closely resembles the individual’s aortic arch morphology from the 3DCT and pre-creates the fenestration according to the position of the branch, making it a semicustom-made method. Additionally, the unique stabilizer function of the Najuta thoracic stent graft enables its precise deployment without the need for cardiac arrest using drugs or rapid pacing (13). Therefore, the Najuta thoracic stent graft system is expected to closely follow the bending and twisting of the aortic arch, thus reducing endoleak and brain complications by simplifying the procedure. Deployment from Zone 0 is also expected to reduce type Ia endoleak from the lesser curvature of the aortic arch caused by the bird-beak configuration (Figures 3A,B).

**Figure 4:**
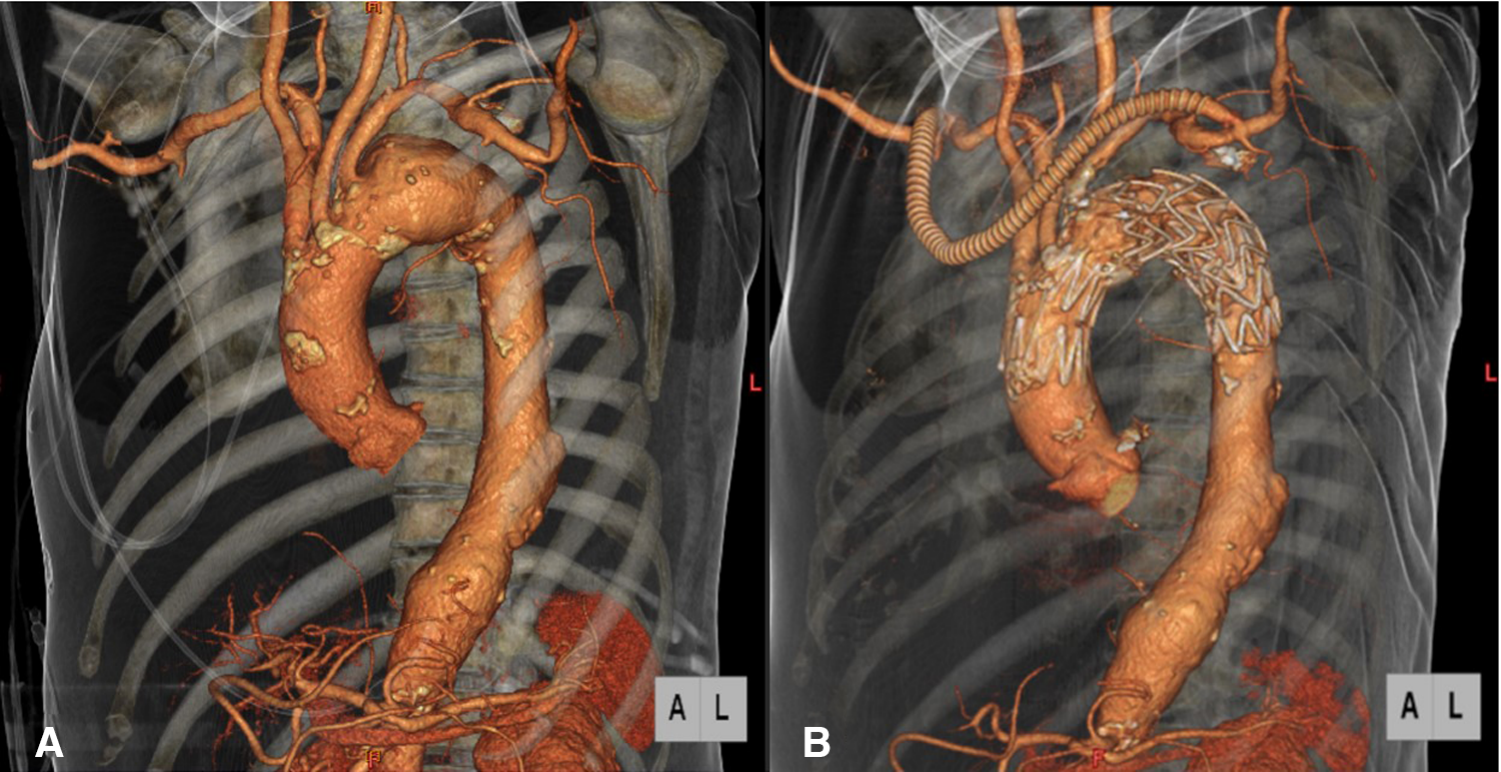
Preoperative CT angiography showed a distal arch aneurysm which involved the left subclavian artery (**A**). Postoperative CT angiography showed patent debranching bypass and exclusion of the aneurysm with the Najuta thoracic stent graft system and Valiant Captivia, with no bird-beak phenomenon in the ascending aorta (**B**).

Regarding the treatment strategy for the 20 patients in our series, 11 patients (55.0%) were treated with two stent grafts, 10 of whom received the distal stent grafts made by other companies, and 2 of the 20 patients (10.0%) were treated with three stent grafts. The reason for this treatment strategy is that in cases where the proximal neck length is <20 mm, the proximal neck length is extended with the Najuta thoracic stent graft after ensuring a certain degree of proximal neck sealing by a peripherally inserted stent graft as a scaffold. This enhances the therapeutic effect. It is considered that the Najuta thoracic stent graft, which has a weaker radial force, receives blood pressure and expands in Zone 0. The bird-beak phenomenon is less likely to occur than in debranching TEVAR using a stent graft manufactured by other companies (Figures 4A,B).

## Conclusion

The treatment of arch and distal arch aortic aneurysms using the Najuta thoracic stent graft system showed acceptable perioperative and short-term clinical outcomes. The insertion of a tube-type stent graft as a scaffold on the peripheral site utilizes the best features of the Najuta thoracic stent graft by extending the landing zone to Zone 0. This avoids the bird-beak phenomenon and catastrophic aortic events during the follow-up. TEVAR using the Najuta thoracic stent graft system may be a potential treatment option for arch and distal arch aortic aneurysms. Global accumulation of treatment experience and careful follow-up are mandatory to build firm evidence regarding the effectiveness of this novel and promising device.

## Data Availability

The original contributions presented in the study are included in the article/Supplementary Material, further inquiries can be directed to the corresponding author.
